# Helicobacter pylori Biofilm Involves a Multigene Stress-Biased Response, Including a Structural Role for Flagella

**DOI:** 10.1128/mBio.01973-18

**Published:** 2018-10-30

**Authors:** Skander Hathroubi, Julia Zerebinski, Karen M. Ottemann

**Affiliations:** a Department of Microbiology and Environmental Toxicology, University of California, Santa Cruz, California, USA; University of Illinois at Chicago; Humboldt-Universität zu Berlin; Vanderbilt University

**Keywords:** *Helicobacter pylori*, biofilm, flagella, stress, metabolism, RNA-seq, transcriptome, stress response, transcriptomics

## Abstract

Helicobacter pylori has an impressive ability to persist chronically in the human stomach. Similar characteristics are associated with biofilm formation in other bacteria. The H. pylori biofilm process, however, is poorly understood. To gain insight into this mode of growth, we carried out comparative transcriptomic analysis between H. pylori biofilm and planktonic cells, using the mouse-colonizing strain SS1. Optimal biofilm formation was obtained with a low concentration of serum and 3 days of growth, conditions that caused both biofilm and planktonic cells to be ∼80% coccoid. Transcriptome sequencing (RNA-seq) analysis found that 8.18% of genes were differentially expressed between biofilm and planktonic cell transcriptomes. Biofilm-downregulated genes included those involved in metabolism and translation, suggesting these cells have low metabolic activity. Biofilm-upregulated genes included those whose products were predicted to be at the cell envelope, involved in regulating a stress response, and surprisingly, genes related to formation of the flagellar apparatus. Scanning electron microscopy visualized flagella that appeared to be a component of the biofilm matrix, supported by the observation that an aflagellated mutant displayed a less robust biofilm with no apparent filaments. We observed flagella in the biofilm matrix of additional H. pylori strains, supporting that flagellar use is widespread. Our data thus support a model in which H. pylori biofilm involves a multigene stress-biased response and that flagella play an important role in H. pylori biofilm formation.

## Abstract (precis)

Biofilms, communities of bacteria that are embedded in a hydrated matrix of extracellular polymeric substances, pose a substantial health risk and are key contributors to many chronic and recurrent infections. Chronicity and recalcitrant infections are also common features associated with the ulcer-causing human pathogen H. pylori. However, relatively little is known about the role of biofilms in H. pylori pathogenesis, as well as the biofilm structure itself and the genes associated with this mode of growth. In the present study, we found that H. pylori biofilm cells highly expressed genes related to cell envelope and stress response, as well as those encoding the flagellar apparatus. Flagellar filaments were seen in high abundance in the biofilm. Flagella are known to play a role in initial biofilm formation, but typically are downregulated after that state. H. pylori instead appears to have coopted these structures for nonmotility roles, including a role building a robust biofilm.

## INTRODUCTION

Helicobacter pylori has been coevolving with humans for tens of thousands of years (1). During this time, it has adapted to survive the hostile environment of the stomach and evade the immune system, allowing it to persist for the life of the host (2). H. pylori colonizes gastric epithelial surfaces and within the thin layer of mucus near the cells (3). More recently, H. pylori was found to colonize within gastric glands, repeated invaginations of the gastrointestinal tract, which may provide the bacteria a favorable niche (4, 5). Even though most infections are asymptomatic, H. pylori persistence is considered a major risk factor for gastric and duodenal ulcers, gastric mucosa-associated lymphoid tissue (MALT) lymphoma, and gastric adenocarcinoma (6). H. pylori infections remain difficult to treat, and when left untreated (7), 1% to 2% progress to gastric cancer (8, 9).

H. pylori possesses several mechanisms to escape the challenging environment of the stomach, where the pH is around 2. These include urease production, flagellar motility, and chemotaxis, which are all required for the initial and sustained colonization of the gastric epithelial surface (10). Urease catalyzes the hydrolysis of urea, which is abundant in the stomach, into bicarbonate and ammonia and thus raises the pH to near neutral (10). pH elevation decreases the viscoelastic properties of mucus gel and improves the motility of H. pylori, which can then swim away from the lumen to reach safer niches, including those close to the gastric epithelial surface (11). H. pylori forms microcolonies at the cell surface *in vitro* (12, 13) as well as within gastric glands (5). This microcolony mode of growth may be consistent with the bacteria being in a biofilm growth mode.

Biofilms are dense aggregates of microorganisms attached to a surface and embedded in an extracellular polymeric matrix (14). In contrast with the other mode of bacterial growth, free-floating or planktonic, biofilm cells tend to be more tolerant toward antimicrobials and host immune responses (14, 15). Biofilms are also frequently associated with chronic disease, including pneumonia in cystic fibrosis patients, Lyme disease, and chronic otitis media (16–18). In those chronic diseases, biofilm growth is considered to be a survival strategy used by pathogens to escape antimicrobial therapies, to avoid clearance by the immune system, and to persist for the lifetime of the host.

Chronicity and recalcitrant infections are also common features associated with H. pylori (19). Yet, the role of biofilm growth in promoting H. pylori persistence is still not clear (20). The first suggestion of biofilm formation by H. pylori during colonization of the human gastric mucosa was found using biopsies and scanning electron microscopy (SEM) analysis (20–22). These studies demonstrated that gastric biopsy samples from H. pylori-positive patients showed dense layers of bacteria aggregated and attached to the mucosal surface. The bacteria were consistent in appearance with H. pylori, with cells in both the spiral and coccoid morphologies. The same bacterial-appearing structures were absent in H. pylori*-*negative patients; however, there has not yet been conclusive evidence showing that H. pylori forms a biofilm *in vivo*.

H. pylori has been well documented to form a biofilm *in vitro*. The first report of *in vitro* biofilm formation by H. pylori was described to occur in clinical, laboratory, and mouse-adapted strains and was observed at the air-liquid interface on glass coverslips when the bacteria were grown in brucella broth (BB) supplemented with slightly lower than normal fetal bovine serum (FBS) (23). The biofilms were mainly composed of coccoid bacteria, with a minority of spiral and rod-shaped cells (23). In subsequent reports, scientists analyzed the extracellular polymeric substance (EPS) of H. pylori biofilms and found proteomannans, lipopolysaccharide (LPS)-related structures, extracellular DNA (eDNA), proteins, and outer membrane vesicles (24, 25).

Additionally, biofilm cells have been shown to exhibit high resistance *in vitro* to clarithromycin, which is one of the common antibiotics used to treat H. pylori infection (26). The MIC and the minimum bactericidal concentration (MBC) were increased by 16- and 4-fold, respectively, in the biofilm cells compared to planktonic ones (26). However, despite the growing evidences of H. pylori biofilm formation both *in vitro* and *in vivo* (20–22, 24), little is known about the genes involved in biofilm formation. We thus sought to characterize H. pylori biofilm and investigate global transcriptional changes during biofilm formation, with a particular focus on H. pylori strain SS1 because it is able to colonize mice and thus will be able to serve as a model for biofilm formation *in vivo*.

## RESULTS

### Biofilm formation and growth condition.

H. pylori strain SS1 has been extensively used as a murine model of H. pylori infection. H. pylori SS1 biofilms, however, are difficult to detect when the bacteria are grown in the standard nutrient-rich media routinely used for H. pylori culture. A previous study reported that H. pylori biofilm formation was significantly dependent on the growth media used (27). We thus, evaluated the ability of the H. pylori SS1 strain to form a biofilm using the crystal violet biofilm assay, as well as bacteria grown under various growth conditions that included different growth media, incubation times, and concentrations of serum. H. pylori SS1 and the other H. pylori strains used in this study are listed in Table 1.

**TABLE 1:** Strains used in this study

H. pylori strain	Knockout strain no.	Description/genotype	Reference or source
SS1			
WT		Wild-type strain	62; J. O’Rourke
Δ*fliM* mutant	KO1064	Δ*fliM::cat*	This study (allele published in reference 41)
Δ*motB* mutant	KO536	Δ*motB2*	65
G27			
WT		Wild-type strain	66; N. Salama, Fred Hutchison Cancer Research Center, Seattle, WA
*motB* mutant	KO493	*motB*::*aphA3*	This study
*flgS* mutant	KO688	*flgS*::Tn*cat*	67
*fliA* mutant	KO689	*fliA*::Tn*cat*	67

**FIG 1:**
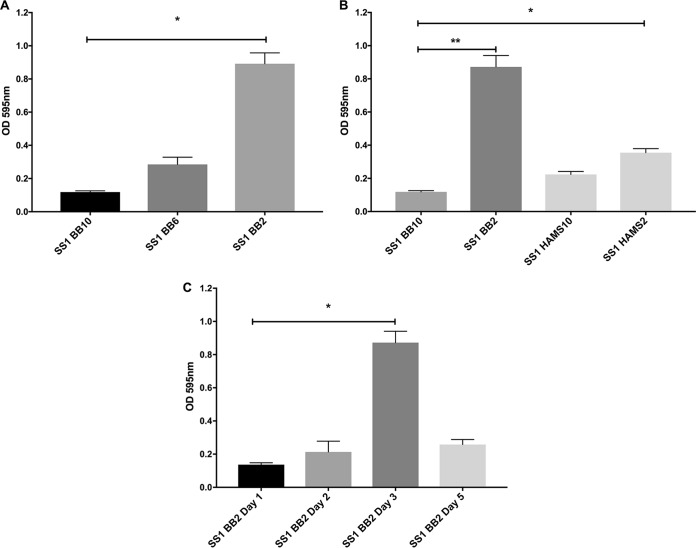
H. pylori SS1 forms robust biofilms after 3 days of growth in BB2. H. pylori strain SS1 was grown in the indicated media, and biofilm formation was assessed by crystal violet absorbance at 595 nm. (A) H. pylori SS1 was grown for 3 days in BB media supplemented with different concentrations of FBS (BB10, 10%; BB6, 6%; and BB2, 2%). (B) H. pylori SS1 was grown for 3 days in BB media or Ham’s F-12 supplemented with 10% (HAMS10) or 2% (HAMS2) FBS. (C) H. pylori SS1 was grown in BB medium supplemented with 2% FBS, and biofilm formation was evaluated at different time points. Experiments were performed three independent times with at least 6 technical replicates for each. Statistical analysis was performed using ANOVA (*, *P < *0.05; and **, *P < *0.01).

Using brucella broth (BB) medium supplemented with 10% FBS (BB10), the condition usually used for H. pylori liquid growth, no biofilm was detected. We therefore explored smaller amounts of serum, as these have been reported elsewhere to promote adhesion of H. pylori strain 26695 (28) and biofilm formation of H. pylori reference strain ATCC 43629 and H. pylori clinical strains 9/10 (27). While only a slight biofilm was observed when H. pylori SS1 strain was grown with BB supplemented with 6% FBS, a pronounced biofilm (*P <* 0.01) was detected in BB supplemented with 2% FBS (BB2) (Fig. 1A). The H. pylori growth rate was slightly reduced in BB2 compared with BB10, which suggests that the increase of biofilm formation was not due to increased growth (data not shown). Ham’s F-12 similarly only supported biofilm formation with low percentages of FBS (Fig. 1B). Further experiments identified that 3 days of growth in BB2 led to the greatest amount of biofilm (Fig. 1C). These results thus, suggest that BB medium supplemented with 2% serum and growth for 3 days is an optimal condition for studying H. pylori SS1 biofilm formation.

### Biofilm characterization.

**FIG 2:**
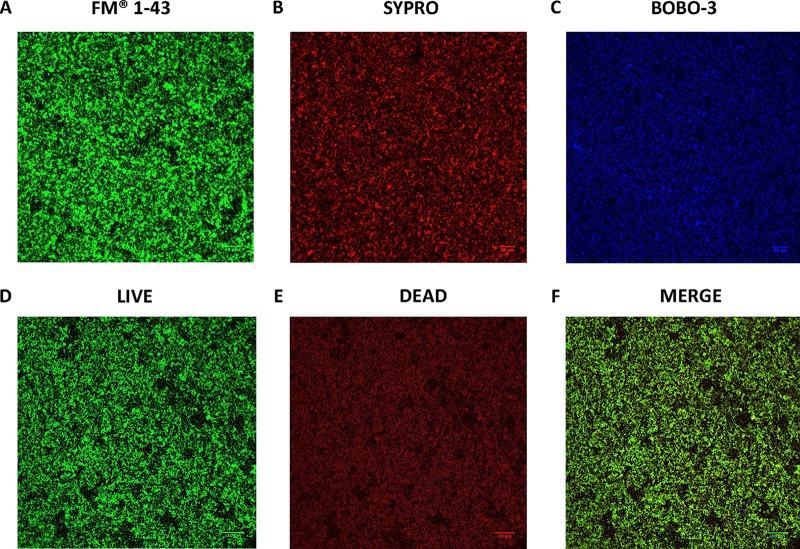
Confocal scanning laser microscopy (CSLM) images of H. pylori SS1 biofilm. Shown are representative CSLM images of 3-day-old SS1 biofilms grown in BB2 and stained with (A) FM 1–43 to stain total bacterial cells, (B) SYPRO RUBY to stain extracellular proteins, (C) BOBO-3 to stain extracellular DNA, and (D to F) live-dead staining with live cells represented by the green fluorescent SYTO 9 and dead/damaged cells represented by the red fluorescent propidium. Scale bar = 30 µm.

To confirm and extend the results obtained with the crystal violet biofilm assay, biofilms of H. pylori SS1 were visualized by confocal laser scanning microscopy (CLSM) and staining with FilmTracer FM 1–43, a dye that fluoresces once inserted into the cell membrane. After 3 days of growth in BB2, we observed a thick bacterial biomass that nonhomogeneously covered the surface, consistent with a well-developed biofilm (Fig. 2). Using z-stack images, the thickness of the H. pylori SS1 biofilm was determined to be 11.64 ± 2.63 μm^3^/μm (see Movie S1 in the supplemental material). As expected, H. pylori SS1 grown in BB10 did not form a biofilm that could be visualized by CLSM (data not shown).

10.1128/mBio.01973-18.1 MOVIE S1 Three-dimensional (3D) view of H. pylori biofilm grown for 3 days. Bacteria were stained with FilmTracer FM 1–43 and observed by CLSM. Download Movie S1, MOV file, 4.4 MB. Copyright © 2018 Hathroubi et al. 2018 Hathroubi et al. This content is distributed under the terms of the Creative Commons Attribution 4.0 International license.

To further characterize the EPS that composed the SS1 biofilm matrix, BOBO-3 and FilmTracer SYPRO Ruby biofilm matrix stains were used to stain extracellular DNA (eDNA) and extracellular proteins, respectively, as described previously (29, 30). Both of these molecules extensively stained the biofilm EPS, consistent with the idea that the H. pylori SS1 biofilm matrix contains significant amounts of eDNA and extracellular proteins (Fig. 2B and C). Because the same molecules have been detected in other H. pylori strains, these results suggest that the H. pylori EPS is typically composed of eDNA and proteins (24, 31).

We also performed live-dead staining with the FilmTracer LIVE/DEAD biofilm viability kit, to define whether the biofilm cells were alive or dead. This approach revealed a subpopulation of dead or damaged cells, stained red, that appear to be homogeneously distributed within the live biofilm cells, which stained green (Fig. 2D to F). This result suggests that the H. pylori biofilm contains both live and dead cells.

**FIG 3:**
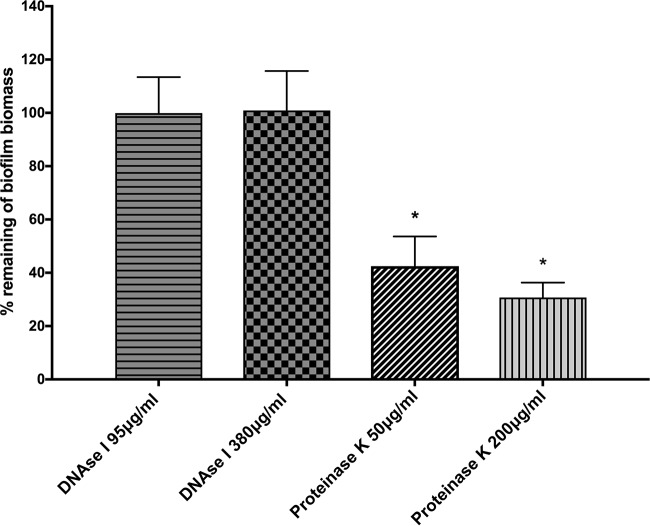
Effect of enzymatic treatments on preformed biofilms. H. pylori SS1 was allowed to form biofilms for 3 days in BB2. The medium was then removed and replaced with either fresh medium or medium containing DNase I or proteinase K. Cells were reincubated for 24 h and then analyzed for the remaining biofilm using the crystal violet assay. The data shown here represent the percentage of remaining biofilm compared to the untreated control. Experiments were performed three times independently with at least 8 technical replicates for each. Statistical analysis was performed using ANOVA (*, *P < *0.01 compared to the untreated control).

To determine the importance of extracellular proteins and eDNA in the biofilm matrix of H. pylori SS1, we employed enzymatic treatment using DNase I and proteinase K. Proteinase K treatment significantly dispersed preformed biofilms (*P < *0.01) (Fig. 3). H. pylori preformed biofilms were, however, resistant to DNase treatments. These data suggest that DNA may play only a minor role in the biofilm matrix; however, extracellular proteins likely play an important role in the biofilm architecture of H. pylori, as has been reported in other H. pylori strains (24, 31). These results suggest that many H. pylori strains, including SS1, use a protein-based biofilm matrix.

### Transcriptomic profiling of biofilm versus planktonic cells.

**FIG 4:**
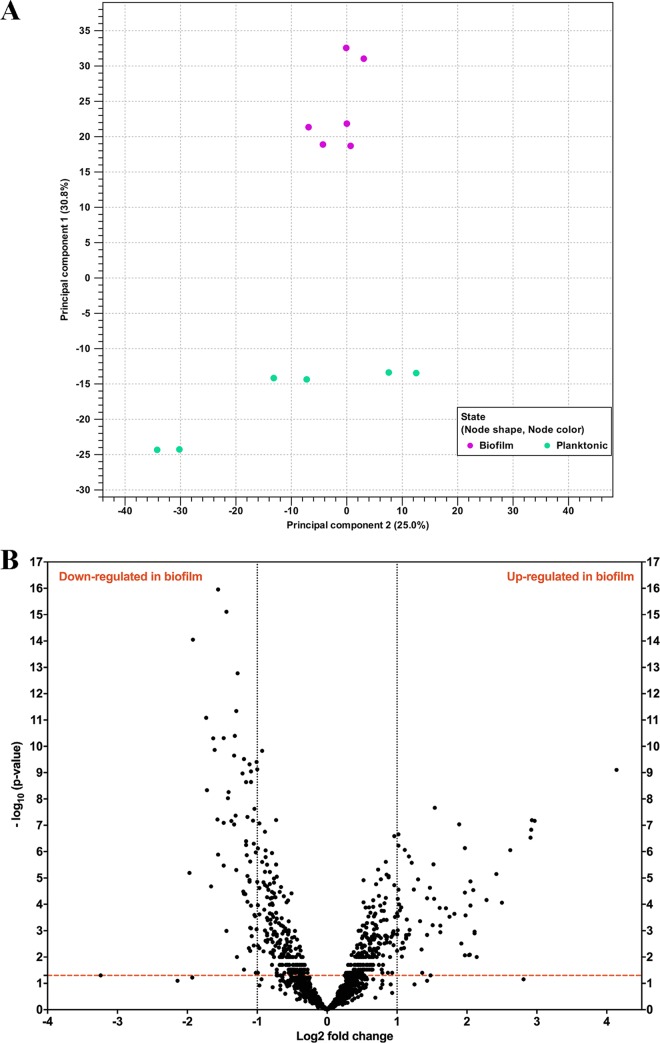
Biofilm-grown cells and planktonic cells show distinct transcriptional profiles. (A) Principal-component analysis (PCA) of gene expression obtained by RNA-seq between biofilm (*n* = 3) and planktonic (*n* = 3) populations. (B) Volcano plot of gene expression data. The *y* axis is the negative log_10_ of *P* values (a higher value indicates greater significance), and the *x* axis is the log_2_ fold change in difference in abundance between two population (positive values represent the upregulated genes in biofilm, and negative values represent downregulated genes). The dashed red line shows where *P* = 0.01, with points above the line having a *P* value of *<*0.01 and points below the line having a *P* value of *>*0.01.

**FIG 5:**
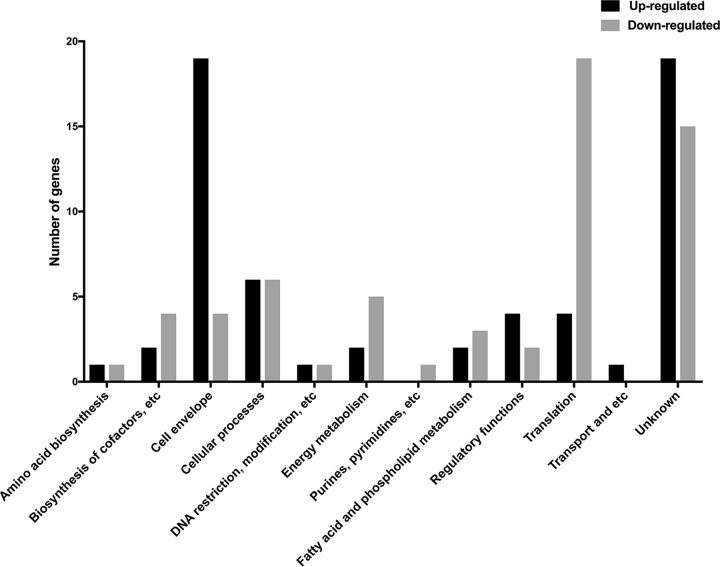
Functional classification of genes differentially expressed in H. pylori SS1 biofilm. Black and gray bars represent upregulated and downregulated genes, respectively, that were significantly differentially expressed (*P <* 0.01 and log_2_ fold change of >1 or <−1) between H. pylori biofilm and planktonic populations.

**FIG 6:**
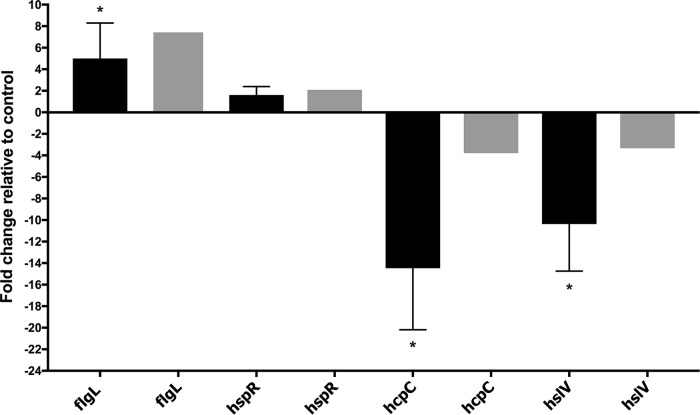
qPCR validation of the transcription of selected differentially expressed genes. The data indicate the fold change in expression of genes in H. pylori biofilm cells compared to planktonic cells. Fold changes in gene expressions were calculated after normalization of each gene with the constitutively expressed gene control *gapB*. Bars represent the mean and error bars the standard error of the mean. Black and gray bars represent qPCR and RNA-seq results, respectively. Statistical analyses were performed using threshold cycle (2^−ΔΔ^*^CT^*) values, and all results with an asterisk were statistically significant (*P <* 0.01).

To gain insight into the genes involved in H. pylori biofilm growth, we performed a transcript profiling experiment using transcriptome sequencing (RNA-seq). For this experiment, we grew H. pylori SS1 in BB2 in six-well plates for 3 days and collected the free-floating planktonic cells and the bottom-attached biofilm ones from the same wells. We collected RNA from three biological replicates grown on 2 separate days. A total of 10 to 20 million reads per sample were generated by RNA-seq. These reads were then mapped to the H. pylori SS1 complete reference genome (32) and revealed a clear clustering of the biofilm-grown cells in a distinct population compared to the planktonic ones (Fig. 4A). This transcriptomic analysis showed that 122 of 1,491 genes (8.18%) were significantly differentially expressed (*P <* 0.01 and log_2_ fold change of >1 or <−1) between H. pylori biofilm and planktonic populations (Fig. 4B and Fig. 5). Sixty-one genes were significantly upregulated in biofilm cells compared to their planktonic counterparts, while another 61 were significantly upregulated in planktonic cells (Table 2 and Table 3). To validate the results obtained by this RNA-seq, the relative abundance of selected RNA transcripts was quantified by quantitative reverse transcription-PCR (qRT-PCR). Using this approach, we detected the same gene expression trend between qRT-PCR and RNA-seq, thus, validating our results (Fig. 6). Below we discuss the most prominent of these genes and what they suggest about the H. pylori biofilm growth state.

**TABLE 2:** Upregulated genes in *H. pylori* SS1 biofilm using RNA-seq analysis, grouped by functional role categories^*a*^

Locus	Putative identification	Fold change^*b*^
Cell envelope		
*flgL* (HPYLSS1_00284)	Flagellar hook-associated protein 3	7.64
*flgK* (HPYLSS1_01062)	Flagellar hook-associated protein 1	6.16
*flgM* (HPYLSS1_01066)	Anti-sigma 28 factor	3.94
*flaG* (HPYLSS1_00586)	Polar flagellin G	3.93
*flaB* (HPYLSS1_00110)	Flagellin B	3.52
*flgE1* (HPYLSS1_00464)	Flagellar hook protein 1	3.07
*flgB* (HPYLSS1_01503)	Flagellar basal body rod protein	2.37
*fliL* (HPYLSS1_00526)	Flagellar protein of unknown function	2.25
*fliK* (HPYLSS1_00653)	Flagellar hook-length control protein	2.14
*fliD* (HPYLSS1_00585)	Flagellar hook-associated protein 2	2
*lpxB* (HPYLSS1_00467)	Lipid A-disaccharide synthase	3.06
*lptB* (HPYLSS1_00622)	Lipopolysaccharide export system ATP-binding	2.54
*mltD* (HPYLSS1_01517)	Membrane-bound lytic murein transglycosylase D precursor	2
*pgdA* (HPYLSS1_00299)	Peptidoglycan deacetylase	2.1
HPYLSS1_00450	Membrane protein	3.78
HPYLSS1_01378	Outer membrane protein *homD*	3.26
HPYLSS1_01113	Putative outer membrane protein	2.91
HPYLSS1_01021	Outer membrane protein *homC*	2.69
HPYLSS1_01469	Putative outer membrane protein	2.52
Cellular processes		
*cagE* (HPYLSS1_00705)	Type IV secretion system protein VirB4/DNA transfer	2.46
*cagW* (HPYLSS1_00718)	*cag* pathogenicity island protein CagW (*cag10*)	2.31
*cagL* (HPYLSS1_00710)	*cag* pathogenicity island protein CagL (*cag18*)	2.02
*recR* (HPYLSS1_00636)	Recombination protein RecR	3.7
HPYLSS1_00410	DNA polymerase I	2.08
HPYLSS1_01332	CMP-*N*-acetylneuraminate-β-galactosamide	2.2
Regulatory functions		
*hrcA* (HPYLSS1_00106)	Heat-inducible transcription repressor HrcA	4.84
*hspR* (HPYLSS1_00407)	Putative heat shock protein HspR	2.09
*crdR* (HPYLSS1_01312)	Two-component response regulator CrdR	2.03
*rsfS* (HPYLSS1_01340)	Ribosomal silencing factor S	2.16
Translation		
*ansA* (HPYLSS1_00615)	Putative l-asparaginase	2.11
*cbpA* (HPYLSS1_00408)	Curved DNA-binding protein	2.19
HPYLSS1_00252	Chaperone protein ClpB	2.7
HPYLSS1_01332	CMP-*N*-acetylneuraminate-β-galactosamide	2.2
Amino acid biosynthesis		
*porC* (HPYLSS1_01050)	Pyruvate synthase subunit PorC	2.02
Fatty acid and phospholipid metabolism		
*acpS* (HPYLSS1_00527)	Holo-[acyl-carrier-protein] synthase	2.89
*fenF* (HPYLSS1_00085)	Malonyl CoA-acyl carrier protein transacylase	2.26
Biosynthesis of cofactors, prosthetic groups, and carriers		
*thiE* (HPYLSS1_00492)	Thiamine-phosphate synthase	4.13
*salL* (HPYLSS1_00914)	Adenosyl-chloride synthase	2.87
DNA restriction, modification, recombination, and repair		
HPYLSS1_00696	Restriction endonuclease	2.19
Transport and binding proteins		
*metI* (HPYLSS1_01522)	d-Methionine transport system permease protein	2.18
HPYLSS1_00805	Putative ABC transporter ATP-binding protein	2.02
Energy metabolism		
*ansA* (HPYLSS1_00615)	Putative l-asparaginase	2.11
HPYLSS1_00772	Pyrroloquinoline quinone biosynthesis protein	7.5
Hypothetical proteins		
HPYLSS1_00605	Hypothetical protein/putative GTPase dynamin	17.63
HPYLSS1_00355	Hypothetical protein	7.82
HPYLSS1_01063	Hypothetical protein	7.58
HPYLSS1_00488	Hypothetical protein	5.65
HPYLSS1_01091	Hypothetical protein	5.34
HPYLSS1_00197	Hypothetical protein	4.4
HPYLSS1_00109	Hypothetical protein	4.25
HPYLSS1_00933	Hypothetical protein	3.91
HPYLSS1_01183	Hypothetical protein	3.37
HPYLSS1_00583	Hypothetical protein	3.07
HPYLSS1_00404	Hypothetical protein	2.85
HPYLSS1_01474	Hypothetical protein	2.77
HPYLSS1_00984	Hypothetical protein	2.56
HPYLSS1_01009	Hypothetical protein	2.26
HPYLSS1_01271	Hypothetical protein	2.05
HPYLSS1_00558	Hypothetical protein	2.04
HPYLSS1_01019	Hypothetical protein	2.03
HPYLSS1_00777	Hypothetical protein	2.01
HPYLSS1_00529	Hypothetical protein	2

a Upregulation was determined as a cutoff ratio of ≥1 log_2_ fold change and *P* value of <0.05.

b Fold change represents the difference in gene expression between biofilm (*n* = 3) and planktonic (*n* = 3) populations.

**TABLE 3:** Downregulated genes in *H. pylori* SS1 biofilm using RNA-seq analysis, grouped by functional role categories^*a*^

Locus	Putative identification	Fold change^*b*^
Cell envelope		
*murB* (HPYLSS1_01344)	UDP-*N*-acetylenolpyruvoylglucosamine reductase	−2.52
*fliM* (HPYLSS1_00401)	Flagellar motor switch protein FliM	−2.25
*fliI* (HPYLSS1_01346)	Flagellum-specific ATP synthase	−2.16
*yohD-1* (HPYLSS1_00775)	Inner membrane protein YohD	−2.14
Cellular processes		
*mreB* (HPYLSS1_01316)	Rod shape-determining protein MreB	−2.95
*ureA* (HPYLSS1_00068)	Urease subunit α	−2.28
*groEL* (HPYLSS1_00013)	60-kDa chaperonin	−2.17
*hcpC* (HPYLSS1_01039)	Cysteine-rich protein HcpC	−3.78
*cmmA* (HPYLSS1_01486)	Polymer-forming cytoskeletal family protein	−2.58
*typA* (HPYLSS1_00442)	GTP-binding protein	−2.02
Regulatory functions		
*hslV* (HPYLSS1_00733)	ATP-dependent protease subunit	−3.33
HPYLSS1_00758	Putative TrmH family tRNA/rRNA	−2.24
Translation		
*rplR* (HPYLSS1_01253)	50S ribosomal protein L18	−3.09
*rpsE* (HPYLSS1_01252)	30S ribosomal protein S5	−3.06
*rpsG* (HPYLSS1_01140)	30S ribosomal protein S7	−2.52
*rpsC* (HPYLSS1_01262)	30S ribosomal protein S3	−2.49
*rpsK* (HPYLSS1_01246)	30S ribosomal protein S11	−2.44
*rplW* (HPYLSS1_01266)	50S ribosomal protein L23	−2.29
*rplN* (HPYLSS1_01258)	50S ribosomal protein L14	−2.29
*rplD* (HPYLSS1_01267)	50S ribosomal protein L4	−2.23
*rplB* (HPYLSS1_01265)	50S ribosomal protein L2	−2.22
*rplF* (HPYLSS1_01254)	50S ribosomal protein L6	−2.21
*rpmF* (HPYLSS1_00189)	50S ribosomal protein L32	−2.14
*rpsD* (HPYLSS1_01245)	30S ribosomal protein S4	−2.12
*rplS* (HPYLSS1_01093)	50S ribosomal protein L19	−2.09
*rplE* (HPYLSS1_01256)	50S ribosomal protein L5	−2.07
*rplV* (HPYLSS1_01263)	50S ribosomal protein L22	−2.02
*rpmG* (HPYLSS1_01151)	50S ribosomal protein L33	−2.02
*fusA* (HPYLSS1_01139)	Elongation factor G	−2.23
*tufA* (HPYLSS1_01152)	Elongation factor Tu	−2.14
*yigZ* (HPYLSS1_01411)	Elongation factor	−2.05
Amino acid biosynthesis		
*trpB* (HPYLSS1_01240)	Tryptophan synthase β chain	−2.68
Fatty acid and phospholipid metabolism		
*acpP_2* (HPYLSS1_00944)	Acyl carrier protein	−2.96
*plsX* (HPYLSS1_00190)	Phosphate acyltransferase	−2.71
*scoB* (HPYLSS1_00895)	3-Oxoacid coenzyme A-transferase, subunit B	−2.11
Biosynthesis of cofactors, prosthetic groups, and carriers		
*birA* (HPYLSS1_01084)	Bifunctional ligase/repressor BirA	−3.29
*ribH* (HPYLSS1_00002)	6,7-Dimethyl-8-ribityllumazine synthase	−2.47
*folK* (HPYLSS1_00396)	2-Amino-4-hydroxy-6-hydroxymethyldihydropteridine pyrophosphokinase	−2.13
*ggt* (HPYLSS1_01061)	γ-Glutamyl transpeptidase	−2.06
DNA restriction, modification, recombination, and repair		
HPYLSS1_00145	Recombinase A	−2.46
Energy metabolism		
*atpC* (HPYLSS1_01075)	ATP synthase ε chain	−2.78
*atpE* (HPYLSS1_01164)	ATP synthase subunit c	−2.48
*nifU* (HPYLSS1_00210)	NifU-like protein	−2.15
*adhA* (HPYLSS1_00)	Alcohol dehydrogenase	−2.12
*mdaB* (HPYLSS1_00836)	Modulator of drug activity B	−2.05
Purine, pyrimidine, nucleosides, and nucleotide		
*pyrD_2* (HPYLSS1_01468)	Dihydroorotate dehydrogenase B [NAD(+)], catalytic subunit	−2.13
Hypothetical proteins		
HPYLSS1_00188	Hypothetical protein	−3.92
HPYLSS1_00885	Hypothetical protein	−3.15
HPYLSS1_00325	Hypothetical protein/putative β-lactamase	−2.95
HPYLSS1_00036	Hypothetical protein/putative nucleoid-associated protein	−2.79
HPYLSS1_01458	Hypothetical protein	−2.79
HPYLSS1_01225	Hypothetical protein	−2.71
HPYLSS1_00259	Hypothetical protein	−2.66
HPYLSS1_01321	Hypothetical protein	−2.44
HPYLSS1_01060	Hypothetical protein	−2.32
HPYLSS1_00657	Hypothetical protein	−2.27
HPYLSS1_01143	Hypothetical protein	−2.21
HPYLSS1_00296	Hypothetical protein/putative F_o_F_1_-ATPase subunit	−2.2
HPYLSS1_00945	Hypothetical protein	−2.16
HPYLSS1_00057	Hypothetical protein	−2.03
HPYLSS1_00569	Hypothetical protein	−2.07

a Downregulation was determined as a cutoff ratio of ≤−1 log_2_ fold change and *P* value of <0.05.

b Fold change represents the difference in gene expression between biofilm (*n* = 3) and planktonic (*n* = 3) populations.

Our data suggest that biofilm cells may be less metabolically active than planktonic cells, based on the decreased expression of several genes involved in translation and ribosomal structure (Fig. 5 and Table 3). Similarly, genes involved in metabolism, biosynthesis of cofactors, and urease were also downregulated (Fig. 5 and Table 3).

We found evidence that biofilm cells experience a stressful environment. Indeed, genes coding for several stress response-related proteins, such as *hrcA*, *hspR*, *crdR*, *recR*, and *pgdA*, were upregulated in biofilm cells (Table 2). The *hspR* and *hrcA* genes code for transcriptional repressor proteins belonging to the heat shock protein family, and both were upregulated in biofilm cells. The *crdR* gene, which encodes a copper-related transcriptional response regulator, was also upregulated in H. pylori biofilm cells. Several transcripts encoding oxidative stress resistance were similarly upregulated in biofilm cells. These included *recR*, a gene encoding a DNA recombination protein, as well as *pgdA*, which encodes a peptidoglycan deacetylase. These have both been previously associated with oxidative stress in H. pylori (33).

We found that the ATP-dependent protease *hslV* gene was among the most downregulated genes in H. pylori biofilm (Table 3). Although the HSlV protein has not yet been studied in the context of H. pylori biofilms, the orthologous Escherichia coli HslV protease has been previously associated with biofilm dispersal (34).

Our data suggest that biofilm cells may be less virulent in some ways, but more in others. Transcripts coding for some H. pylori virulence, colonization, or immunogenic factors were low in biofilm cells, including the UreA subunit of urease, the GroEL chaperone, and the HcpC cysteine-rich protein. These have each been shown to play roles in colonization or promoting inflammatory gene expression (35–37). On the other hand, only three genes carried within the cytotoxin-associated gene pathogenicity island (*cag*PAI) (38, 39), *cagL*, *cagW*, and *cagE*, were significantly highly expressed in biofilm cells of H. pylori. These genes are in separate operons (38) and encode *cag*PAI protein CagL/Cag18, an integrin binding protein at the Cag pilus tip, *cag*PAI protein CagW/Cag10, and type IV secretion system protein CagE/VirB4, both part of the inner membrane protein transfer complex (39).

Many genes related to the cell envelope were upregulated in biofilm cells (Fig. 5). Indeed, genes coding for proteins involved in lipopolysaccharide synthesis, such as *lpxB*, which encodes a lipid A disaccharide synthase, and *lptB*, which encodes a lipopolysaccharide export system ATPase, were upregulated in biofilm cells (Table 2). Numerous transcripts encoding cytoplasmic and outer membrane proteins were also elevated in biofilm cells (i.e., *homC*, *homD*, and HPYLSS1_00450) (Table 2).

Interestingly, the majority of the upregulated cell envelope genes in biofilm cells encoded flagellar structure and biosynthesis proteins, such as *flgL*, *flgK*, *fliD*, and *flgE*, which encode flagellar hook-associated proteins (Table 2). Two known or putative flagellin genes, *flaB* and *flaG*, were also upregulated in the biofilms (Table 2). These data suggested the intriguing idea that flagella might play a role in H. pylori biofilm.

### Flagella are present and play a structural role in H. pylori biofilms.

**FIG 7:**
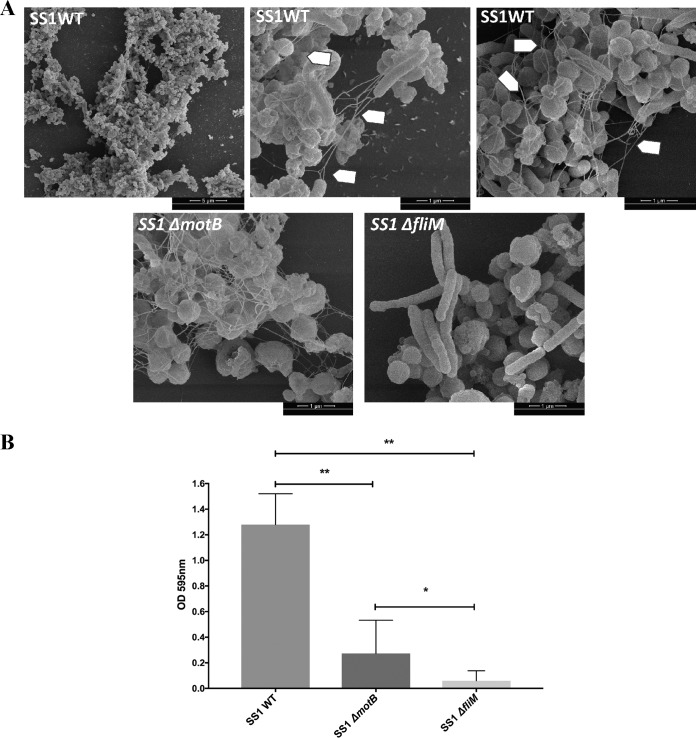
Flagella play integral roles in H. pylori biofilms. (A) Scanning electron microscope (SEM) images of biofilms formed by H. pylori wild-type SS1 (SS1 WT), the isogenic nonmotile but flagellated Δ*motB* mutant (SS1 Δ*motB*), and the isogenic aflagellated Δ*fliM* mutant (SS1 Δ*fliM*). Arrows indicate flagella. (B) Quantification of biofilm formation by the H. pylori SS1 wild type and Δ*motB* and Δ*fliM* mutants. Strains were grown in BB2 medium for 3 days, followed by biofilm evaluation using the crystal violet assay. Experiments were performed three times independently with 6 to 9 technical replicates for each. Statistical analysis was performed using ANOVA (**, *P < *0.01; and *, *P < *0.05).

The transcriptomic data above suggested that flagellar components are upregulated in the biofilm cells, so we used SEM to gain insights into the biofilm architecture of H. pylori. This analysis demonstrated three-dimensional structures composed of bacterial cells adherent to one another and to the surface (Fig. 7A). Biofilms contained mainly coccoid cells along with some rod-shaped cells (Fig. 7A), as described previously for H. pylori biofilms (20–23). Compared to planktonic populations, the proportions of both morphologies were similar at ∼80% coccoid cells (data not shown).

Interestingly, extensive networks of bundles of filaments were visible in the biofilms. In some cases, these appeared to be connected to the bacterial pole, as would be expected for flagella (Fig. 7A, arrowheads). We measured the dimensions of the filaments to see if they were consistent in size with flagella. The width and the length measured 20 to 30 nm and 3 to 4 μm, respectively, and were in agreement with those reported previously for H. pylori flagella (40). These data, especially when combined with transcriptomics, suggested these structures could be flagella. We therefore analyzed a mutant strain that lacks a key component of the flagellar basal body, FliM, and is aflagellated (41). SEM analysis of the Δ*fliM* mutant showed a complete loss of flagella (Fig. 7A). This mutant displayed significantly less biofilm biomass (Fig. 7B), but we were able to find few microcolonies. Within these microcolonies, the filaments were completely lacking (Fig. 7A). These results suggest that these filaments are flagella and, furthermore, that flagella and/or motility is important for biofilm formation.

To further dissect the roles of motility and flagella in biofilm formation, we analyzed biofilm formation in a nonmotile but flagellated strain created by disruption of the motor protein MotB. As reported previously, this mutant still expresses flagella (Fig. 7A). Biofilm formation, however, was severely impaired compared to that in the wild-type strain, which suggests that a lack of motility might contribute to the biofilm defect. However, the Δ*motB* mutant produced significantly more biofilm than the Δ*fliM* mutant, suggesting that the flagellar structure even in the absence of motility also contributes to biofilm formation in H. pylori.

**FIG 8:**
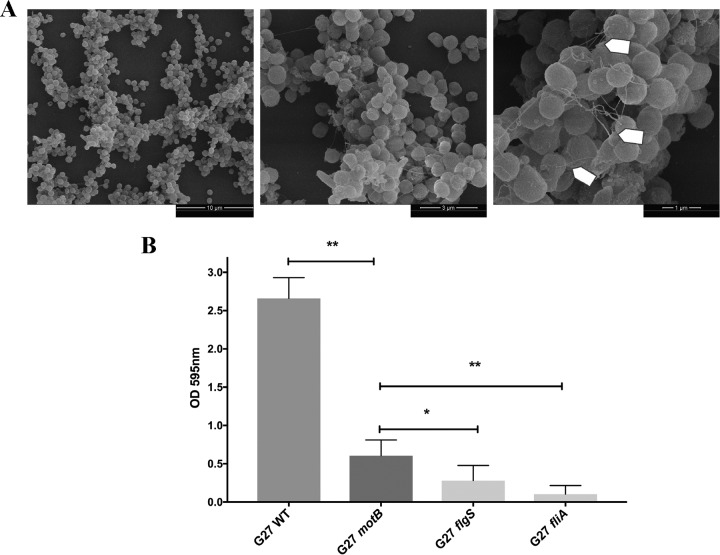
H. pylori G27 biofilm contains structurally important flagella. (A) Scanning electron microscope (SEM) images of wild-type G27 H. pylori biofilms. Arrows indicate flagella. (B) Quantification of biofilm formation by the H. pylori G27 wild type (WT), the nonmotile flagellated *motB* mutant, the nonmotile *fliA* mutant that is reported to have either truncated flagella or no flagella, and the aflagellated and nonmotile *flgS* mutant. Biofilms were evaluated using the crystal violet assay. Experiments were performed 2 times independently with at least 6 technical replicates for each. Statistical analysis was performed using ANOVA (**, *P < *0.01; and *, *P < *0.05).

To examine whether other strains of H. pylori similarly use flagella in biofilms, we imaged the biofilm of H. pylori strain G27 and similar flagellar mutants to those used above. Wild-type H. pylori G27 biofilm cells also contained filaments consistent with flagella (Fig. 8A). As with strain SS1, mutants lacking flagella (*flgS or fliA*) formed very weak biofilms, while strains that had flagella but no motility (*motB*) retained partial biofilm formation (Fig. 8B).

Taken together, these data suggest that flagella are produced by H. pylori when in a biofilm and appear to play roles in addition to simple motility, promoting biofilm integrity by holding cells together and to the surface.

## DISCUSSION

In this report, we present the first transcriptomic characterization of the H. pylori biofilm. This study demonstrated clearly distinct expression profiles between planktonic and biofilm cells. The biofilm cells were characterized by low metabolic activity and triggering of several stress responses. Among the upregulated genes in the biofilm cells, we found several genes associated with cell membrane proteins, outer membrane proteins, and stress response, as well as, surprisingly, genes related to the flagellar apparatus. SEM analysis confirmed that flagella are present in a mature H. pylori biofilm and appear to play a role in maintaining solid biofilm structures. This result was somewhat surprising, as typically flagella are proposed to be turned off during the sessile biofilm growth mode (42–44). Recent work, however, discussed below has suggested that flagella in E. coli biofilms may play a structural role. Our studies with H. pylori thus build on an emerging theme that flagella are not always turned off in mature biofilms and indeed may play important functions in biofilm structure.

To gain insights into the mechanisms behind the biofilm formation in H. pylori, we used RNA sequencing and carried out a comparative transcriptomic analysis between biofilm cells and those in the planktonic state. Using this approach, we observed that 8.18% of genes were significantly differentially expressed between biofilm and planktonic cells, similar to what has been reported in other bacterial systems (43, 45, 46). In our experimental design, we compared a static biofilm mode of growth, where attached cells adhered to the bottom of the wells, with planktonic nonattached cells in the same wells. This approach was used to maintain the same growth conditions as much as possible between biofilm and planktonic samples and likely contributed to the relatively small number of differentially expressed genes. However, since biofilm formation is a dynamic process with frequent switching between planktonic and biofilm modes occurring frequently, we likely have some contamination between the biofilm and planktonic populations. Therefore, our method may have missed some genes that are expressed in either population.

One of the findings from our transcriptomic analysis was that several flagellar protein transcripts were significantly elevated in the biofilm. Notably, these were not for the entire flagellum but instead specific genes encoding the rod, hook, and filament. Specifically, we saw biofilm cell overexpression of genes encoding the FlgB rod protein, the FlgE flagellar hook protein, the FlgK and FlgL hook-filament junction proteins, the FliK hook length control protein, and two flagellins (FlaB and the putative flagellin encoded by *flaG*). Notably absent was the gene for the major flagellin FlaA and genes for the motor and stator. We also saw the upregulation of *flgM*, which encodes an anti-sigma factor that interacts with flagellar sigma factor FliA and therefore would be expected to decrease expression of *flaA* (47).

Historically, flagella have been typically viewed as important only for initial biofilm attachment and later cell dispersion (44, 48). In fact, it has often been suggested that genes encoding flagella are turned off in mature biofilms (42–44). However, other reports have shown that some microbes express flagella during all stages of biofilm development, not only during the attachment and dispersion processes (49, 50). In E. coli, several flagellar biosynthesis genes were induced in mature biofilms, and around 20 flagellar genes were regulated throughout all stages of biofilm development and not simply turned off (49). E. coli flagella were proposed to have a structural role along with other matrix components (i.e., eDNA and extracellular proteins), acting to cement and hold cells together and to the surface (50, 51). Our data furthermore showed that aflagellated mutants are poor biofilm formers, supporting that these filaments could play a structural role. Taken together, these findings suggest that flagella of H. pylori may play a structural role during biofilm formation to help bacteria attach to each other and to surfaces.

Interestingly, we found that the HspR and HrcA transcriptional repressor proteins are upregulated in biofilm cells. These proteins had previously been shown to positively correlate with flagellar expression (52), providing candidate regulatory proteins that function in biofilm cells. HspR and HrcA belong to the heat shock protein family and have been shown to respond to heat shock temperature conditions, although the nature of their “true” signal is not yet clear (52, 53). A previous comparative transcriptomic analysis of wild-type H. pylori along with Δ*hspR,* Δ*hrcA*, and double mutants revealed a set of 14 genes that were negatively regulated and 29 genes that were positively regulated by these transcriptional regulators (52). The regulated genes include those for chaperones, urease enzyme activity, adhesion to epithelial cells, and flagella. Interestingly, among the 29 positively regulated genes, nearly half (14) encoded flagellar proteins, including the *flgM*, *flaG*, *fliD*, *flgK*, *flgB*, *flgE*, and *fliK* transcripts we identified here. Thus, our data suggest that biofilm conditions activate expression of HrcA and HspR, which in turn upregulate a subset of flagellar genes.

Experiments suggest that HrcA and HspR regulators do not directly regulate the flagellar genes (52). However, they do directly repress expression from several promoters, including those upstream of the *groESL*, *hrcA-grpE-dnaK*, and *cbpA-hspR-hp1026* operons. These gene products encode the major chaperones of H. pylori (52–54). Heat shock conditions relieve the repression and allow expression of these operons. Consistent with elevated expression of HspR and HrcA, we found the genes coding for the heat shock protein GroEL to be downregulated in biofilm. Our data suggest that some yet-to-be-determined conditions occurring during biofilm formation trigger the expression of HspR and HcrA regulators.

Other genes associated with stress responses were also upregulated in biofilm cells, including the *pgdA* and *recR* genes. These genes encode a peptidoglycan deacetylase and DNA recombination protein, respectively. RecR has been shown to be involved in repairing in DNA double-strand breaks induced by oxidative stress (55), and the *recR* mutant was highly sensitive to DNA-damaging agents and oxidative stress and had a reduced ability to colonize mouse stomachs (55). *pgdA* has been reported to be highly induced by oxidative stress (33, 56). Upregulation of oxidative stress genes has previously been reported in biofilm cells of other organisms, including E. coli (57), Pseudomonas aeruginosa (42), Neisseria gonorrhoeae (58), and Clostridium perfringens (48).

As reported for other microorganisms, H. pylori biofilm cells have altered metabolism, typically thought to be associated with the restricted availability of nutrients (48, 59). H. pylori biofilm cells were characterized by downregulation of the expression of multiple genes involved in metabolism and translation, including *atpC*, *atpE*, and *nifU*, and several ribosomal protein genes. This low-metabolism phenotype seems not be related simply to the presence of coccoid cells but rather to the microenvironment generated during biofilm formation since the proportions of rods and coccoid forms did not differ between planktonic and biofilm populations.

H. pylori biofilm cells may also actively block the translational machinery, as suggested by the upregulation of the gene encoding RsfS, a ribosomal silencing factor. This protein was previously described in E. coli and Mycobacterium tuberculosis to slow or block the translation machinery during stationary phase and/or nutrition deficiency stress (60). It interacts with the 50S large ribosomal subunit, prevents its association with the 30S ribosomal submit, and thus blocks formation of functional ribosomes (60). Whether it functions similarly in H. pylori remains to be determined.

These observations that biofilm cells may have decreased translation are relevant because at least two of the main antibiotics used to treat H. pylori infection, clarithromycin and tetracycline, inhibit the 50S and 30S ribosomal subunits, respectively. Thus, these antibiotics may have less impact on biofilm cells. In fact, recent *in vitro* studies have shown that clarithromycin is 4- to 16-fold less effective on H. pylori biofilm cells than on planktonic ones (26, 61).

Taken together, our study has shown that H. pylori biofilm cells display a distinct transcriptomic profile compared to their planktonic counterparts. Lower metabolism and stress responses, likely associated with the microenvironment generated in the H. pylori biofilm, could be determinants of antimicrobial tolerance and involved in the persistence and survival of H. pylori. However, the upregulated and downregulated genes identified in this study are not specific for biofilm cells, and stress response genes have been previously observed under other conditions when both planktonic and biofilm cells were exposed to various stresses. Therefore, our data do not support the existence of a biofilm-specific genetic program. Additionally, our data show that flagellar filaments are upregulated in biofilm cells and form an integral part of the biofilm matrix. Indeed, H. pylori cells without flagella form weak biofilms. These results thus contribute to correcting the idea that flagella are only involved during the first and last steps of biofilm formation and instead support their importance throughout the biofilm process.

## MATERIALS AND METHODS

### Bacterial strain and growth conditions.

H. pylori Sydney strain 1 (SS1) (62) and all other H. pylori strains used in this study are listed in Table 1. Strains were grown on Columbia horse blood agar (CHBA) containing 0.2% β-cyclodextrin, 10 μg/ml vancomycin, 5 μg/ml of cefsulodin, 2.5 U/ml polymyxin B, 5 μg/ml trimethoprim, and 8 μg/ml amphotericin B (all chemicals from Thermo Fisher or Gold Biotech). Cultures were grown under microaerobic conditions (5% O_2_ and 10% CO_2_) at 37°C. For liquid culture and biofilm assay, H. pylori was grown in brucella broth (Difco) containing 10% heat-inactivated fetal bovine serum (FBS) (BB10 [Gibco/BRL]) with constant shaking under microaerobic conditions. For biofilm formation, several conditions were tested, including brucella broth containing different percentages of FBS (BB2, BB6, and BB10) and Ham’s F-12 (PAA Laboratories GmbH, Pasching, Austria) containing 10% or 2% FBS (HAMS10 and HAMS2, respectively).

### Biofilm assays.

Biofilm formation assays were carried out as described previously, with slight modification (27). H. pylori SS1 was grown overnight in BB10 as described above, diluted to an optical density at 600 nm (OD_600_) of 0.15 with fresh BB10, BB2, BB6, or Ham’s medium as desired, and then used to fill triplicate wells of a sterile 96-well polystyrene microtiter plate (Costar no. 3596). Following static incubation for 1, 2, 3, or 5 days under microaerobic conditions, culture medium was removed by aspiration, and the plate was washed twice with phosphate-buffered saline (PBS). The wells were then filled with 200 μl of crystal violet (0.1% wt/vol), and the plate was incubated for 2 min at room temperature. After removal of the crystal violet solution by aspiration, the plate was washed twice with PBS and dried for 20 min at room temperature. To visualize biofilms, 200 μl of ethanol (70% vol/vol) was added to the wells, and the absorbance at 590 nm was measured.

### Biofilm dispersion assays.

To evaluate the composition of SS1 biofilm matrix, we assessed the response of preformed biofilms to different enzymatic treatments. DNase I and proteinase K (both from Sigma-Aldrich) were used to target extracellular DNA and extracellular proteins, respectively. Biofilms were grown as described above, and after 3 days of growth, the old media were replaced by fresh media containing different concentrations of DNase I (from 380 μg/ml to 95 μg/ml) or proteinase K (from 200 μg/ml to 50 μg/ml). The cells were then incubated for a further 24 h. Control wells were exposed to media without enzyme. After treatments, the biofilm was stained with crystal violet as described above. Results are presented a percentage of the untreated control.

### Confocal laser scanning microscopy.

Biofilms of H. pylori SS1 were prepared as described above using BB2; however, for confocal laser scanning microscopy (CLSM), μ-Slide 8-well glass bottom chamber slides (ibidi, Germany) were used instead of 96-well microtiter plates. Three-day-old biofilms were stained with FilmTracer FM 1–43 (Invitrogen), BOBO-3 (Invitrogen), Filmtracer SYPRO Ruby biofilm matrix stain (Invitrogen), or the FilmTracer LIVE/DEAD biofilm viability kit (Invitrogen) according to the manufacturer’s instructions. Stained biofilms were visualized by CLSM with an LSM 5 Pascal laser scanning microscope (Zeiss) and images were acquired using Imaris software (Bitplane). Biomass analysis of biofilm was carried using FM 1–43-stained z-stack images (0.1-μm thickness) obtained by CLSM from randomly selected areas. The biomass of biofilms was determined using COMSTAT (63).

### RNA extraction and library construction.

Biofilms of H. pylori SS1 were grown in 6-well plates (Costar) in BB2 as described above. After 3 days of incubation, medium containing nonattached planktonic bacteria (the planktonic fraction) was removed by pipetting, and the cells were harvested by centrifugation and washed twice with PBS. The attached bacteria, representing the biofilm fraction, were washed twice with PBS to remove any remaining planktonic cells. Attached cells were scrapped off the plate using a cell scraper. Both planktonic and biofilm fractions were subject to total RNA extraction using the TRIzol Max bacterial enhancement kit (Ambion, Life Technology, Carlsbad, CA) as described by the manufacturer. RNA was further purified and concentrated using an RNeasy kit (Qiagen). rRNA was removed using the RiboZero magnetic kit (Illumina). Sequencing libraries were generated using NEBNext Ultra Directional RNA library prep kit for Illumina (NEB, USA). cDNA library quality and amount were verified using the Agilent Bioanalyzer 2100 system (Agilent Technologies, CA) and then sequenced using Illumina NextSeq Mid-Output (University of California Davis Genome Center).

### Transcriptomic analysis.

RNA-seq data were analyzed using CLC Genomics Workbench (version 11.0; CLC Bio, Boston, MA). After adapters were trimmed, forward- and reverse-sequenced reads generated for each growth state (biofilm versus planktonic, with three biological replicates for each condition) were mapped against the SS1 reference genome (32) to quantify gene expression levels for each experimental condition. The expression value was measured in reads per kilobase per million mapped reads (RPKM). Genes were considered differentially expressed when the log_2_ fold change was above 1 and the *P* value was less than 0.05.

### Quantitative PCR.

To validate the RNA-seq data, we performed qPCR to quantify the transcription of four differentially expressed genes (two upregulated genes and two downregulated genes). The fold change in gene expression was calculated after normalization of each gene with the constitutively expressed gene *gapB* (64). Primers used for this experiment are listed 5′ to 3′ below: gapB forward, GCCTCTTGCACGACTAACGC, and gapB reverse, CTTTGCTCACGCCGGTGCTT; flgL forward, CAGGCAGCTCATGGATGCGA, and flgL reverse, CGCTGTGCAAGGCGTTTTGA; hspR forward, TAGGCGTGCACCCTCAAACC, and hspR reverse, CGCCCGCTAGATTAACCCCC; hcpC forward, GGGTTTTGTGCTTGGGTGCG, and hcpC reverse, TTCCACCCCCTGCCCTTGAT; and hslV forward, GATTTGCCGGAAGCACTGCG, and hslV reverse, ATCATCGCTTCCAGTCGGCG.

### Construction of H. pylori mutants.

The SS1 Δ*fliM* mutant was created by natural transformation of the SS1 wild type with plasmid pBS-fliM::catmut (40), which replaces most of the *fliM* gene, corresponding to amino acids 1 to 105, with *cat*. The G27 *motB* mutant was created by natural transformation of the G27 wild type with plasmid pKO114K and selection for kanamycin resistance. pKO114K was made as described for pKO114i (65), but instead of insertion of an *aphA3*-*sacB* allele, only an *aphA3* allele was inserted. This allele inserts the *aphA3* gene at the position corresponding to amino acid 113 of 257.

### Scanning electron microscopy.

H. pylori biofilms were grown on glass coverslips (12 mm; Chemglass Life Sciences, Vineland, NJ) by dispersing 4 ml of a culture diluted to an OD of 0.15 in BB2 into wells of a 6-well plate (Costar). The plate was incubated as described above. After 3 days of growth, biofilms formed on the surface of the coverslips and planktonic cells were washed twice with PBS and fixed with 2.5% (wt/vol) glutaraldehyde for 1 h at room temperature. Samples were then dehydrated with a graded ethanol series, critical point dried, sputter coated with ∼20 nm of gold (Hammer IV, Technics, Inc., Anaheim, CA), and imaged in an FEI Quanta 3D Dualbeam scanning electron microscope operating at 5 kV and 6.7 pA.

### Statistical analysis.

Biofilm data were analyzed with GraphPad Prism (version 7.0) software (GraphPad, Inc., San Diego, CA) using one-way analysis of variance (ANOVA) followed by Dunnett’s multiple-comparison test.

## References

[1] Moodley Y, Linz B, Bond RP, Nieuwoudt M, Soodyall H, Schlebusch CM, Bernhoft S, Hale J, Suerbaum S, Mugisha L, van der Merwe SW, Achtman M 2012 Age of the association between *Helicobacter pylori* and man. PLoS Pathog 8:e1002693. doi:10.1371/journal.ppat.1002693. 22589724 PMC3349757

[2] Salama NR, Hartung ML, Muller A 2013 Life in the human stomach: persistence strategies of the bacterial pathogen *Helicobacter pylori*. Nat Rev Microbiol 11:385–399. doi:10.1038/nrmicro3016. 23652324 PMC3733401

[3] Schreiber S, Konradt M, Groll C, Scheid P, Hanauer G, Werling HO, Josenhans C, Suerbaum S 2004 The spatial orientation of *Helicobacter pylori* in the gastric mucus. Proc Natl Acad Sci U S A 101:5024–5029. doi:10.1073/pnas.0308386101. 15044704 PMC387367

[4] Keilberg D, Zavros Y, Shepherd B, Salama NR, Ottemann KM 2016 Spatial and temporal shifts in bacterial biogeography and gland occupation during the development of a chronic infection. mBio 7:e01705-16. doi:10.1128/mBio.01705-16. 27729513 PMC5061875

[5] Howitt MR, Lee JY, Lertsethtakarn P, Vogelmann R, Joubert LM, Ottemann KM, Amieva MR 2011 ChePep controls *Helicobacter pylori* infection of the gastric glands and chemotaxis in the Epsilonproteobacteria. mBio 2:e00098-11. doi:10.1128/mBio.00098-11. 21791582 PMC3143842

[6] Hu Q, Zhang Y, Zhang X, Fu K 2016 Gastric mucosa-associated lymphoid tissue lymphoma and *Helicobacter pylori* infection: a review of current diagnosis and management. Biomark Res 4:15. doi:10.1186/s40364-016-0068-1. 27468353 PMC4962427

[7] Kim SY, Choi DJ, Chung JW 2015 Antibiotic treatment for *Helicobacter pylori*: is the end coming? World J Gastrointest Pharmacol Ther 6:183–198. doi:10.4292/wjgpt.v6.i4.183. 26558152 PMC4635158

[8] Parkin DM 2006 The global health burden of infection-associated cancers in the year 2002. Int J Cancer 118:3030–3044. doi:10.1002/ijc.21731. 16404738

[9] Choi IJ, Kook MC, Kim YI, Cho SJ, Lee JY, Kim CG, Park B, Nam BH 2018 *Helicobacter pylori* therapy for the prevention of metachronous gastric cancer. N Engl J Med 378:1085–1095. doi:10.1056/NEJMoa1708423. 29562147

[10] Keilberg D, Ottemann KM 2016 How *Helicobacter pylori* senses, targets and interacts with the gastric epithelium. Environ Microbiol 18:791–806. doi:10.1111/1462-2920.13222. 26768806

[11] Celli JP, Turner BS, Afdhal NH, Keates S, Ghiran I, Kelly CP, Ewoldt RH, McKinley GH, So P, Erramilli S, Bansil R 2009 *Helicobacter pylori* moves through mucus by reducing mucin viscoelasticity. Proc Natl Acad Sci U S A 106:14321–14326. doi:10.1073/pnas.0903438106. 19706518 PMC2732822

[12] Tan S, Tompkins LS, Amieva MR 2009 *Helicobacter pylori* usurps cell polarity to turn the cell surface into a replicative niche. PLoS Pathog 5:e1000407. doi:10.1371/journal.ppat.1000407. 19412339 PMC2669173

[13] Anderson JK, Huang JY, Wreden C, Sweeney EG, Goers J, Remington SJ, Guillemin K 2015 Chemorepulsion from the quorum signal autoinducer-2 promotes *Helicobacter pylori* biofilm dispersal. mBio 6:e00379-15. doi:10.1128/mBio.00379-15. 26152582 PMC4488943

[14] Flemming HC, Wingender J 2010 The biofilm matrix. Nat Rev Microbiol 8:623–633. doi:10.1038/nrmicro2415. 20676145

[15] Hathroubi S, Mekni MA, Domenico P, Nguyen D, Jacques M 2017 Biofilms: microbial shelters against antibiotics. Microb Drug Resist 23:147–156. doi:10.1089/mdr.2016.0087. 27214143

[16] Sapi E, Balasubramanian K, Poruri A, Maghsoudlou JS, Socarras KM, Timmaraju AV, Filush KR, Gupta K, Shaikh S, Theophilus PA, Luecke DF, MacDonald A, Zelger B 2016 Evidence of *in vivo* existence of Borrelia biofilm in borrelial lymphocytomas. Eur J Microbiol Immunol 6:9–24. doi:10.1556/1886.2015.00049. PMC4838982 27141311

[17] Boisvert AA, Cheng MP, Sheppard DC, Nguyen D 2016 Microbial biofilms in pulmonary and critical care diseases. Ann Am Thorac Soc 13:1615–1623. doi:10.1513/AnnalsATS.201603-194FR. 27348071 PMC5059503

[18] Hall-Stoodley L, Hu FZ, Gieseke A, Nistico L, Nguyen D, Hayes J, Forbes M, Greenberg DP, Dice B, Burrows A, Wackym PA, Stoodley P, Post JC, Ehrlich GD, Kerschner JE 2006 Direct detection of bacterial biofilms on the middle-ear mucosa of children with chronic otitis media. JAMA 296:202–211. doi:10.1001/jama.296.2.202. 16835426 PMC1885379

[19] Vakil N, Vaira D 2013 Treatment for *H. pylori* infection: new challenges with antimicrobial resistance. J Clin Gastroenterol 47:383–388. doi:10.1097/MCG.0b013e318277577b. 23388847

[20] Hathroubi S, Servetas SL, Windham I, Merrell DS, Ottemann KM 2018 *Helicobacter pylori* biofilm formation and its potential role in pathogenesis. Microbiol Mol Biol Rev 82:e00001-18. doi:10.1128/MMBR.00001-18. 29743338 PMC5968456

[21] Carron MA, Tran VR, Sugawa C, Coticchia JM 2006 Identification of *Helicobacter pylori* biofilms in human gastric mucosa. J Gastrointest Surg 10:712–717. doi:10.1016/j.gassur.2005.10.019. 16713544

[22] Coticchia JM, Sugawa C, Tran VR, Gurrola J, Kowalski E, Carron MA 2006 Presence and density of *Helicobacter pylori* biofilms in human gastric mucosa in patients with peptic ulcer disease. J Gastrointest Surg 10:883–889. doi:10.1016/j.gassur.2005.12.009. 16769546

[23] Cole SP, Harwood J, Lee R, She R, Guiney DG 2004 Characterization of monospecies biofilm formation by *Helicobacter pylori*. J Bacteriol 186:3124–3132. doi:10.1128/JB.186.10.3124-3132.2004. 15126474 PMC400600

[24] Windham IH, Servetas SL, Whitmire JM, Pletzer D, Hancock REW, Merrell DS 2018 *Helicobacter pylori* biofilm formation is differentially affected by common culture conditions, and proteins play a central role in the biofilm matrix. Appl Environ Microbiol 84:e00391-18. doi:10.1128/AEM.00391-18. 29752266 PMC6029101

[25] Yang FL, Hassanbhai AM, Chen HY, Huang ZY, Lin TL, Wu SH, Ho B 2011 Proteomannans in biofilm of *Helicobacter pylori* ATCC 43504. Helicobacter 16:89–98. doi:10.1111/j.1523-5378.2010.00815.x. 21435085

[26] Yonezawa H, Osaki T, Hanawa T, Kurata S, Ochiai K, Kamiya S 2013 Impact of *Helicobacter pylori* biofilm formation on clarithromycin susceptibility and generation of resistance mutations. PLoS One 8:e73301. doi:10.1371/journal.pone.0073301. 24039906 PMC3765302

[27] Bessa LJ, Grande R, Di Iorio D, Di Giulio M, Di Campli E, Cellini L 2013 *Helicobacter pylori* free-living and biofilm modes of growth: behavior in response to different culture media. APMIS 121:549–560. doi:10.1111/apm.12020. 23237527

[28] Williams JC, McInnis KA, Testerman TL 2008 Adherence of *Helicobacter pylori* to abiotic surfaces is influenced by serum. Appl Environ Microbiol 74:1255–1258. doi:10.1128/AEM.01958-07. 18156334 PMC2258569

[29] Hathroubi S, Hancock MA, Bosse JT, Langford PR, Tremblay YD, Labrie J, Jacques M 2016 Surface polysaccharide mutants reveal that absence of O antigen reduces biofilm formation of *Actinobacillus pleuropneumoniae*. Infect Immun 84:127–137. doi:10.1128/IAI.00912-15. 26483403 PMC4694004

[30] Vogeleer P, Tremblay YDN, Jubelin G, Jacques M, Harel J 2015 Biofilm-forming abilities of Shiga toxin-producing *Escherichia coli* isolates associated with human infections. Appl Environ Microbiol 82:1448–1458. doi:10.1128/AEM.02983-15. 26712549 PMC4771338

[31] Grande R, Di Giulio M, Bessa LJ, Di Campli E, Baffoni M, Guarnieri S, Cellini L 2011 Extracellular DNA in *Helicobacter pylori* biofilm: a backstairs rumour. J Appl Microbiol 110:490–498. doi:10.1111/j.1365-2672.2010.04911.x. 21143715

[32] Draper JL, Hansen LM, Bernick DL, Abedrabbo S, Underwood JG, Kong N, Huang BC, Weis AM, Weimer BC, van Vliet AH, Pourmand N, Solnick JV, Karplus K, Ottemann KM 2017 Fallacy of the unique genome: sequence diversity within single Helicobacter pylori strains. mBio 8:e02321-16. doi:10.1128/mBio.02321-16. 28223462 PMC5358919

[33] Wang G, Maier RJ 2015 A novel DNA-binding protein plays an important role in *Helicobacter pylori* stress tolerance and survival in the host. J Bacteriol 197:973–982. doi:10.1128/JB.02489-14. 25535274 PMC4325109

[34] Hong SH, Lee J, Wood TK 2010 Engineering global regulator Hha of *Escherichia coli* to control biofilm dispersal. Microb Biotechnol 3:717–728. doi:10.1111/j.1751-7915.2010.00220.x. 21255366 PMC3158428

[35] Formichella L, Romberg L, Bolz C, Vieth M, Geppert M, Gottner G, Nolting C, Walter D, Schepp W, Schneider A, Ulm K, Wolf P, Busch DH, Soutschek E, Gerhard M 2013 A novel line immunoassay based on recombinant virulence factors enables highly specific and sensitive serologic diagnosis of *Helicobacter pylori* infection. Clin Vaccine Immunol 20:1703–1710. doi:10.1128/CVI.00433-13. 24006137 PMC3837778

[36] Zhao Y, Yokota K, Ayada K, Yamamoto Y, Okada T, Shen L, Oguma K 2007 *Helicobacter pylori* heat-shock protein 60 induces interleukin-8 via a Toll-like receptor (TLR)2 and mitogen-activated protein (MAP) kinase pathway in human monocytes. J Med Microbiol 56:154–164. doi:10.1099/jmm.0.46882-0. 17244794

[37] Kusters JG, van Vliet AH, Kuipers EJ 2006 Pathogenesis of *Helicobacter pylori* infection. Clin Microbiol Rev 19:449–490. doi:10.1128/CMR.00054-05. 16847081 PMC1539101

[38] Ta LH, Hansen LM, Sause WE, Shiva O, Millstein A, Ottemann KM, Castillo AR, Solnick JV 2012 Conserved transcriptional unit organization of the cag pathogenicity island among *Helicobacter pylori* strains. Front Cell Infect Microbiol 2:46. doi:10.3389/fcimb.2012.00046. 22919637 PMC3417554

[39] Backert S, Tegtmeyer N, Fischer W 2015 Composition, structure and function of the Helicobacter pylori cag pathogenicity island encoded type IV secretion system. Future Microbiol 10:955–965. doi:10.2217/fmb.15.32. 26059619 PMC4493163

[40] O'Rourke J, Bode G 2001 Morphology and ultrastructure, p 53–67. *In* Mobley HLT, Mendz GL, Hazell SL (ed), Helicobacter pylori: physiology and genetics. ASM Press, Washington, DC. 21290711

[41] Lowenthal AC, Hill M, Sycuro LK, Mehmood K, Salama NR, Ottemann KM 2009 Functional analysis of the *Helicobacter pylori* flagellar switch proteins. J Bacteriol 191:7147–7156. doi:10.1128/JB.00749-09. 19767432 PMC2786559

[42] Sauer K, Camper AK, Ehrlich GD, Costerton JW, Davies DG 2002 *Pseudomonas aeruginosa* displays multiple phenotypes during development as a biofilm. J Bacteriol 184:1140–1154. doi:10.1128/jb.184.4.1140-1154.2002. 11807075 PMC134825

[43] Whiteley M, Bangera MG, Bumgarner RE, Parsek MR, Teitzel GM, Lory S, Greenberg EP 2001 Gene expression in *Pseudomonas aeruginosa* biofilms. Nature 413:860–864. doi:10.1038/35101627. 11677611

[44] Guttenplan SB, Kearns DB 2013 Regulation of flagellar motility during biofilm formation. FEMS Microbiol Rev 37:849–871. doi:10.1111/1574-6976.12018. 23480406 PMC3718880

[45] Romero-Lastra P, Sanchez MC, Ribeiro-Vidal H, Llama-Palacios A, Figuero E, Herrera D, Sanz M 2017 Comparative gene expression analysis of *Porphyromonas gingivalis* ATCC 33277 in planktonic and biofilms states. PLoS One 12:e0174669. doi:10.1371/journal.pone.0174669. 28369099 PMC5378342

[46] Beloin C, Valle J, Latour-Lambert P, Faure P, Kzreminski M, Balestrino D, Haagensen JA, Molin S, Prensier G, Arbeille B, Ghigo JM 2004 Global impact of mature biofilm lifestyle on *Escherichia coli* K-12 gene expression. Mol Microbiol 51:659–674. 14731270 10.1046/j.1365-2958.2003.03865.x

[47] Josenhans C, Niehus E, Amersbach S, Horster A, Betz C, Drescher B, Hughes KT, Suerbaum S 2002 Functional characterization of the antagonistic flagellar late regulators FliA and FlgM of *Helicobacter pylori* and their effects on the *H. pylori* transcriptome. Mol Microbiol 43:307–322. doi:10.1046/j.1365-2958.2002.02765.x. 11985711

[48] Charlebois A, Jacques M, Archambault M 2016 Comparative transcriptomic analysis of *Clostridium perfringens* biofilms and planktonic cells. Avian Pathol 45:593–601. doi:10.1080/03079457.2016.1189512. 27207477

[49] Domka J, Lee J, Bansal T, Wood TK 2007 Temporal gene-expression in *Escherichia coli* K-12 biofilms. Environ Microbiol 9:332–346. doi:10.1111/j.1462-2920.2006.01143.x. 17222132

[50] Serra DO, Richter AM, Klauck G, Mika F, Hengge R 2013 Microanatomy at cellular resolution and spatial order of physiological differentiation in a bacterial biofilm. mBio 4:e00103-13. doi:10.1128/mBio.00103-13. 23512962 PMC3604763

[51] Hung C, Zhou Y, Pinkner JS, Dodson KW, Crowley JR, Heuser J, Chapman MR, Hadjifrangiskou M, Henderson JP, Hultgren SJ 2013 *Escherichia coli* biofilms have an organized and complex extracellular matrix structure. mBio 4:e00645-13. doi:10.1128/mBio.00645-13. 24023384 PMC3774191

[52] Roncarati D, Danielli A, Spohn G, Delany I, Scarlato V 2007 Transcriptional regulation of stress response and motility functions in *Helicobacter pylori* is mediated by HspR and HrcA. J Bacteriol 189:7234–7243. doi:10.1128/JB.00626-07. 17693507 PMC2168435

[53] Spohn G, Scarlato V 1999 The autoregulatory HspR repressor protein governs chaperone gene transcription in *Helicobacter pylori*. Mol Microbiol 34:663–674. doi:10.1046/j.1365-2958.1999.01625.x. 10564507

[54] Spohn G, Danielli A, Roncarati D, Delany I, Rappuoli R, Scarlato V 2004 Dual control of *Helicobacter pylori* heat shock gene transcription by HspR and HrcA. J Bacteriol 186:2956–2965. doi:10.1128/JB.186.10.2956-2965.2004. 15126455 PMC400627

[55] Wang G, Lo LF, Maier RJ 2011 The RecRO pathway of DNA recombinational repair in *Helicobacter pylori* and its role in bacterial survival in the host. DNA Repair (Amst) 10:373–379. doi:10.1016/j.dnarep.2011.01.004. 21292567 PMC3062642

[56] Wang G, Maier SE, Lo LF, Maier G, Dosi S, Maier RJ 2010 Peptidoglycan deacetylation in *Helicobacter pylori* contributes to bacterial survival by mitigating host immune responses. Infect Immun 78:4660–4666. doi:10.1128/IAI.00307-10. 20805339 PMC2976313

[57] Schembri MA, Kjaergaard K, Klemm P 2003 Global gene expression in *Escherichia coli* biofilms. Mol Microbiol 48:253–267. doi:10.1046/j.1365-2958.2003.03432.x. 12657059

[58] Falsetta ML, Steichen CT, McEwan AG, Cho C, Ketterer M, Shao J, Hunt J, Jennings MP, Apicella MA 2011 The composition and metabolic phenotype of *Neisseria gonorrhoeae* biofilms. Front Microbiol 2:75. doi:10.3389/fmicb.2011.00075. 21833322 PMC3153042

[59] Castro J, Franca A, Bradwell KR, Serrano MG, Jefferson KK, Cerca N 2017 Comparative transcriptomic analysis of *Gardnerella vaginalis* biofilms vs. planktonic cultures using RNA-seq. NPJ Biofilms Microbiomes 3:3. doi:10.1038/s41522-017-0012-7. 28649404 PMC5460279

[60] Hauser R, Pech M, Kijek J, Yamamoto H, Titz B, Naeve F, Tovchigrechko A, Yamamoto K, Szaflarski W, Takeuchi N, Stellberger T, Diefenbacher ME, Nierhaus KH, Uetz P 2012 RsfA (YbeB) proteins are conserved ribosomal silencing factors. PLoS Genet 8:e1002815. doi:10.1371/journal.pgen.1002815. 22829778 PMC3400551

[61] Bugli F, Palmieri V, Torelli R, Papi M, De Spirito M, Cacaci M, Galgano S, Masucci L, Paroni Sterbini F, Vella A, Graffeo R, Posteraro B, Sanguinetti M 2016 *In vitro* effect of clarithromycin and alginate lyase against *Helicobacter pylori* biofilm. Biotechnol Prog 32:1584–1591. doi:10.1002/btpr.2339. 27535356

[62] Lee A, O'Rourke J, De Ungria MC, Robertson B, Daskalopoulos G, Dixon MF 1997 A standardized mouse model of *Helicobacter pylori* infection: introducing the Sydney strain. Gastroenterology 112:1386–1397. doi:10.1016/S0016-5085(97)70155-0. 9098027

[63] Heydorn A, Nielsen AT, Hentzer M, Sternberg C, Givskov M, Ersboll BK, Molin S 2000 Quantification of biofilm structures by the novel computer program COMSTAT. Microbiology 146:2395–2407. doi:10.1099/00221287-146-10-2395. 11021916

[64] Collins KD, Hu S, Grasberger H, Kao JY, Ottemann KM 2018 Chemotaxis allows bacteria to overcome host-generated reactive oxygen species that constrain gland colonization. Infect Immun. doi:10.1128/IAI.00878-17. PMC5913845 29507083

[65] Ottemann KM, Lowenthal AC 2002 *Helicobacter pylori* uses motility for initial colonization and to attain robust infection. Infect Immun 70:1984–1990. doi:10.1128/IAI.70.4.1984-1990.2002. 11895962 PMC127824

[66] Censini S, Lange C, Xiang Z, Crabtree JE, Ghiara P, Borodovsky M, Rappuoli R, Covacci A 1996 cag, a pathogenicity island of *Helicobacter pylori*, encodes type I-specific and disease-associated virulence factors. Proc Natl Acad Sci U S A 93:14648–14653. doi:10.1073/pnas.93.25.14648. 8962108 PMC26189

[67] Salama NR, Shepherd B, Falkow S 2004 Global transposon mutagenesis and essential gene analysis of *Helicobacter pylori*. J Bacteriol 186:7926–7935. doi:10.1128/JB.186.23.7926-7935.2004. 15547264 PMC529078

